# Love as harm reduction: fighting AIDS and stigma in Vietnam

**DOI:** 10.1186/1477-7517-6-34

**Published:** 2009-12-03

**Authors:** Dan Small

**Affiliations:** 1 Department of Anthropology, University of British Columbia, Vancouver, Canada; 2 Director, PHS Community Services Society, Vancouver, Canada

## Abstract

In the summer of 2009, I visited a humble orphanage for children with HIV/AIDS in Vietnam. Here, like many parts in the world, the very existence of marginalized people with stigmatized illness is hidden away. Relegated to the shadows of society, these children lacked something more fundamental than housing, shelter, nutrition and medications. They lacked families to love and care for them unconditionally. One might think it self-evident that a visit to an orphanage for children with HIV would be profound, but the profundity wasn't where I expected to find it. It was in how the children had created their own family, loving each other like brothers and sisters, and the way the priest who operated the shelters was more than a Father, he was a dad to dozens of children. This is an account of love as harm reduction in the Mai Tam orphanage in Ho Chi Minh City.

## For Bé Hiền

The Mai Tam orphanage and two other shelters in Ho Chi Minh City are operated under the direction of a Catholic priest, Father Toai Dinh Toai in the Archdiocese of Ho Chi Minh City. The first is home to 50 children and 14 mothers with HIV and AIDS. It is known as the Mai Tam shelter. The second provides shelter for 26 children and 12 mothers living with HIV. The third is a hospice where there are currently 16 children in the doorway of death. The priest who operates the shelter is a young man with gentleness in his eyes and a welcoming smile. When I walked with him from room to room, the children reached to touch him like they might a loving mother or father. As a father myself, the love between the children and their collective dad was unmistakable.

Ensuring that the children have shelter is no small task. The program is controversial in Vietnam and there is tremendous difficulty finding a permanent place where children with HIV are welcome. When I visited, the priest and the children were illegally "squatting" in a home after being served eviction by the owner of the home. Despite visits from the police at the request of the landlord, Fr. Toai was holding out until there was a new home for the orphans. Fortunately, a parishioner had donated half of her yard for a shelter and the money had been raised to build a permanent home.

Beyond being a priest, Fr. Toai has undergone training to become a physician's assistant. This allows him to go beyond overseeing the children's shelter and psychosocial needs to managing their health care (including anti-retroviral treatment). Each day, he begins at 6:30 am as he visits the shelters one after another. His day ends at 10:00 each evening after visiting the children's hospice. He has the help of nuns who provide schooling and nurses that visit during the week. Mothers with HIV living in the shelter also help with the care of the children.

Whenever possible, Fr. Toai attempts to reconnect the children with any surviving family members. This is extremely difficult and requires educational tenacity as he works to overcome stigma, fear and lack of knowledge about HIV. He also tries to reintegrate them into mainstream society. This is an enormous challenge given that children with HIV are not even welcome in schools in Ho Chi Minh City. As the school year began in 2009, parents of unaffected children forced officials, who willingly complied, to expel all children living with HIV. The newspapers carried photographs of children with HIV, with tears streaming down their cheeks, ashamedly exiting the schools on the first day of class[1,2].

The priest told me that one of the girls he was trying to integrate into a mainstream school asked him a penetrating question one day: "how come you teach me not to lie, but you tell me to lie at school?" He had instructed her to avoid telling other children that she has HIV when she has to take her antiretroviral medications (ARVs). Instead, he had suggested that she say that she has a heart problem. Clearly, the priest understood that the children do not live in the black and white world of the Ten Commandments.

My impression upon seeing dozens of children in the crowded shelter was a mixture of sadness and rage. My sadness had the same roots as my ire at how we had failed these children. We had failed to put in place the necessary healthcare and social conditions to protect them from contracting HIV. Here these children were, and still are, sequestered from streets of Ho Chi Minh City where their pain might otherwise be publicly acknowledged. They had arrived at the orphanage with the death of their parents or they had been abandoned, deserted and left at the doorstep. Without this modest shelter, they would lack of the basic sustenance necessary to give them the slightest chance at health. But there was something deeply inspiring about this shelter from the cold world.

## Life at Mai Tam

**Figure 1:**
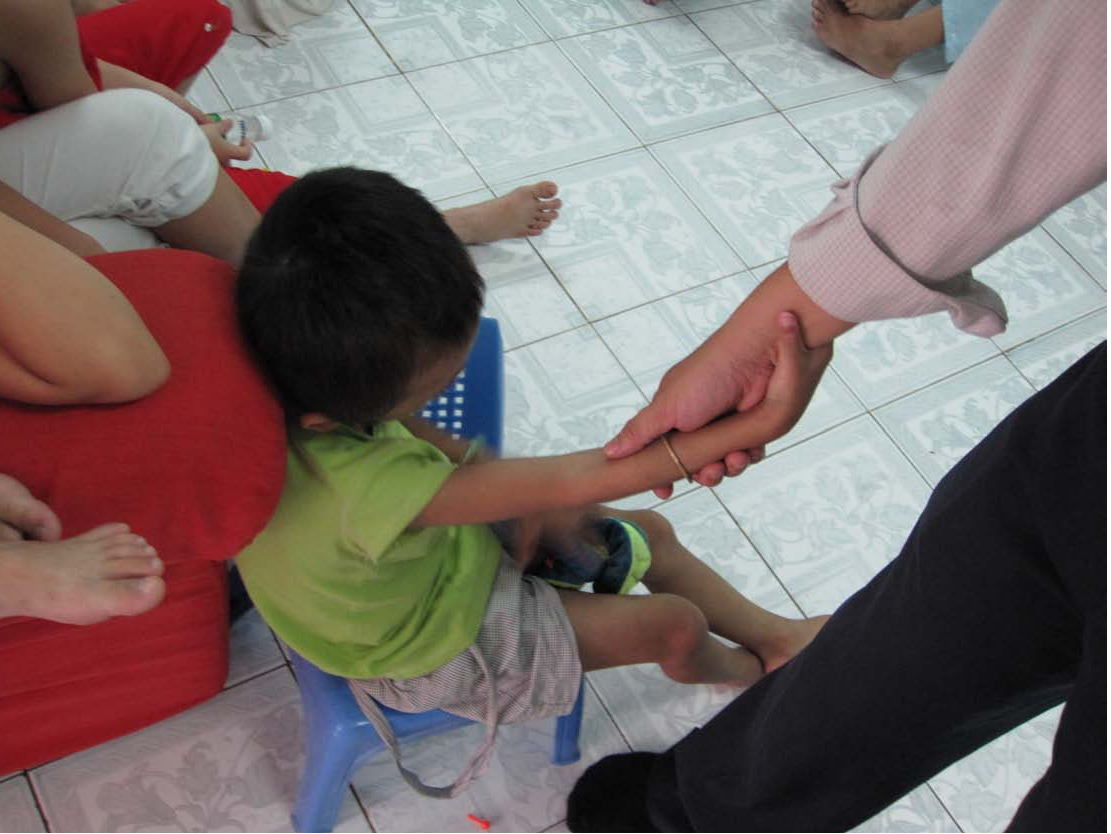
**The Embrace**. A photograph of a loving embrace between a father and son.

At the shelter, the children appeared, for the most part, to be happy. They were drawn to the young priest as though he were their paternal father. I watched as a little one, withered with his illness and as of yet unresponsive to treatment, 7 years old and no more than 30 pounds, weakly reached up from the floor where he sat to touch the Father's hand. The father attentively caressed his little wrist and held onto his little arm with love (see Figure 1). He introduced each child and told his or her story. The young ones signaled their desire to be picked up and held by him by lifting their little arms in the air as he drew near. When he first began speaking, one little boy of about 11 months of age crawled over to the father and tugged at his leg. The Father bent down and picked him up and held him like his own son. One of the young mothers with HIV who live and help in the orphanage reached to hold the baby so that the priest could continue his tour. The little boy clung and protested with big wet tears and cried out for the priest when the young mother took him. It reminded me of my own son at home in Canada, the same age, desperately reaching for me whenever I walk into the room.

The youngest children were in a playroom at naptime when I arrived. They slept on the floor in a room while two young women from the shelter watched over them. There is only one bathroom, with a single shower hose and a bucket, which is used to bath the children. They have donated formula, anti-retroviral HIV medication and some toys. Each child has their own face cloth with their name written on it hanging on a drying rack in the room. The environment, though sparse, appeared to be infused with love, caring and kindness. This love seemed, to me, to be the core ingredient in the healthcare and housing of these children. Without it, I'm convinced; they wouldn't stand a chance to be on the threshold of a successful life.

The youngest child was a small baby of only a few months who was unable to hold her own bottle. She was abandoned in the hospital and was too little to hold her own bottle so one was propped in her mouth. As I looked at her in her small crib, she looked at my face and followed me with her eyes. As one of the young boys began to cry, Fr. Toai walked over to him and asked why he was sad. The little boy told him that another boy had taken his toy. The priest went over to the other child and softly asked for the toy to be given back. The little boy handed the toy to the father and he returned it. The sad boy immediately stopped crying. His "dad" had set the situation right.

## Children teaching kindness

One of the things that was most profound to me was the fact that the children had so much to teach about kindness. It was a kindness that had not been given to them from the wider world from where they had been expelled. As a case in point, a young girl, of about 6 years of age, carried the children around the room, took them from the priest to help him. She would then sit down with them on her lap and stroke their hair and kiss them. One little boy, about 13 months old, provided a lesson in sharing. He was sitting holding his infant bottle and trying to feed another child of the same age. The other child did not want to eat but the little caregiver was persistent. Finally, the little boy took some of the milk: one little baby feeding another. The recipient of the bottle eventually pushed the bottle out of his mouth. The caregiver persistently attempted to feed his little friend again, about 3-dozen tries, each time saying "Uh Uh, *pause*, Uh Uh, *pause*, Uh Uh". The little recipient began to cry and the nurturer stroked his head and consoled him in Vietnamese. Here, were two infants, not yet walking, sharing their scarce food and taking care of each other. When I commented on this, the priest said that the young children had not yet been exposed to the wider world and, as such, have not met people who are so concerned about meeting their own needs. Like brothers and sisters, they had created the love and family none of them had outside the orphanage.

But, despite the lesson they taught about kindness and acceptance, they still have to share the finite amount of parental love available through the father, the nuns and ill mothers. They appeared to me to crave human contact and more than once I felt one of the children brush against my leg and tug on my pant leg to be picked up. When I photographed them, they ran to me to see the digital image. One child, the priest sadly showed us, is isolated because he has tuberculosis. He has to spend all his days and nights, alone, in a single room behind glass windows. I wished that prospective parents would open their hearts a little wider and adopt children with HIV. But, as a rule, they don't.

One of the young children, a boy of about 2 years of age, it turned out, in the fullness of time, was not HIV positive. The priest reported that a Canadian couple attempted to adopt him but have given up after the process appeared too difficult. I asked the Priest if adoption would be ultimately completed: he stated "No, not this time". The little boy slept, without a pillow, on his side on the floor. Earlier a five-year-old girl had held him on her lap and picked him up so that his head was on her shoulder. He proved a little too heavy, while sleeping, and, as a result, his head would roll back and his light hair would hang downwards.

## A powerful history of stigma

The stigma against HIV in Vietnam is powerful. The owner of the home where the majority of orphans lived when I visited wanted the house back. The owner had evicted them despite the fact that they did not have another home where they could live. The Priest has had to raise $200,000 US to obtain a new home: $100,000 for land and another $100,000 for the building. No one would rent them a home because of the stigma of AIDS. Following an update in his homily to his congregation regarding the status of the orphanage, a parishioner, donated half of her lot, with her house still beside it, for the orphanage. As a result of this kindness, the Priest had half of the $200,000 for the new home. Through donations over the years, he was getting closer to the mark. In 2009, he traveled to the U.S. for two weeks to try "raise money". When I asked how he raised money (imagining proposals, pamphlets and PowerPoint presentations) he responded: "I begged in front of churches, like a beggar". He raised $35,000 US asking for help in front of Catholic Churches in Boston.

When I saw all the caring that centred on the work of this one priest, I began to worry about what might happen if he left or became weary. Already knowing the answer, I asked him directly whether there was a succession plan. He told me the answer I expected: there is no one to replace him. I asked how he looked after himself and he said: "that's a very good question". He then told me that he learns so much from the children about kindness and that the children provide him with nourishment that gives him strength.

In the 19^th ^century, people living with leprosy in the Hawaiian Islands were banished to Kalaupapa, an isolated settlement at the top of a steep cliff, in an isolated region in Molokai. At that time, the government offered a bounty for people who turned in lepers and, once discovered, they were sequestered from the wider society because of fear of their condition. Given that relocation was permanent, family members without leprosy often accompanied their loved ones and lived at the leper colony. Over its history, thousands people were exiled there and the population of lepers ranged from several hundred at any time to its most populous of 1,213 in 1890. Approximately 8000 are buried there today[3].

Joseph De Veuster travelled to the Hawaiian islands in 1863 where he was to spend the next 16 years, the remainder of his life, as priest, baker, farmer, physician and carpenter to those stricken with leprosy[4]. He was 33 years old at the time, and after he adopted the name Father Damien upon his ordination in 1864, he volunteered to permanently live amongst the lepers. He, himself, contracted leprosy and died of complications related to the disease on 15 April 1889[3]. He was beatified on 4 June 1995[5]. Today, his memory is also evoked in reference to those people living with AIDS who, like lepers, are feared and stigmatized.

Fr. Toai reminded me of the legend of Fr. Damien caring for the lepers of Molokai. Many healthcare issues, like leprosy or AIDS, exist at a busy intersection of cultural values. In Canada, people with leprosy were quarantined on D'Arcy Island and Bentinck Island in British Columbia between 1894 and 1924 where they were given only the barest of necessities: food and coffins[6]. The afflicted were exiled and left to die on these islands without healthcare despite the fact that leprosy was not acutely contagious. There was also an element of ethnocentrism in that only lepers of Chinese origin received this fate whereas Euro-Canadian lepers, in contrast, enjoyed healthcare services from the nuns of the Hospitalières de Saint-Joseph based in New Brunswick and Quebec [7-9].

Similar to people who are dependent on illicit drugs and those with HIV today, the lepers were often blamed for their disease. They were believed to have brought their disease upon themselves because of morally wrong behaviour. Of course, children with HIV, like persons with leprosy, did not bring this disease upon themselves and their suffering was not self-induced. Fr. Toai is ministering in an isolated community with people who have been expelled and who are considered "not quite" human. Like Fr. Damien, out of necessity, he is creating a parallel world, constructed out of love, with shelter, nutrition, healthcare, education and acceptance.

What's more, the places where more resources exist are not easily available to these children by reason of the stigma associated with their condition. And so, they are also sequestered to their home country. They cannot be easily adopted and brought to places with more universally available resources like my home country Canada by reason of their illness. They are, by sad default, treated as though they are not economically viable, only partially human and therefore unworthy of immigration. Such an application for adoption and immigration would likely be rejected out of hand to protect the taxpayer and public purse from the cost of treating their illness. We will all be broken by serious illness eventually; everyone's body will one day cease to operate. Despite the barriers of stigma, these children may live for decades with sufficient nutrition, shelter, anti-retroviral medications and love. But, regardless, they are considered social lepers.

## Love and kindness: the common thread of caring for others

**Figure 2:**
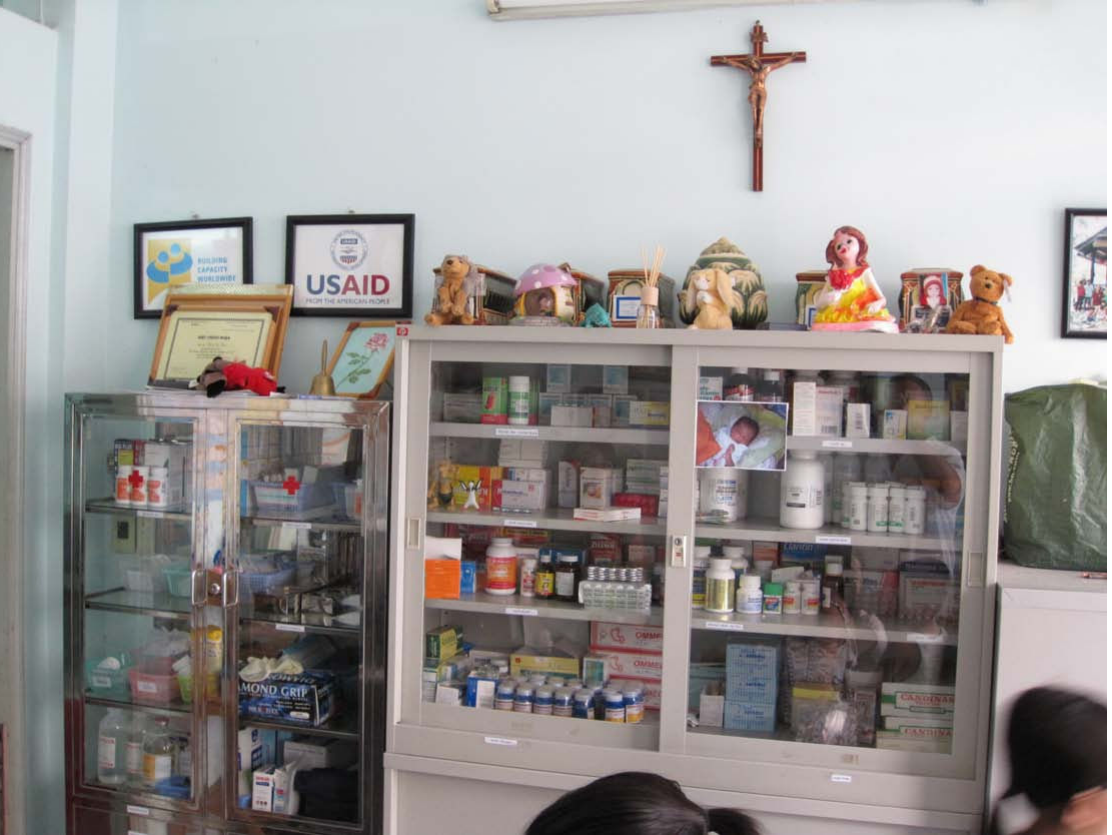
**Urns and ARVs**. A photograph showing antiretroviral medications (ARVs) in a cabinet at the orphanage. The urns of orphaned children lost to AIDS, along with a favourite toy, rest on the top of the cabinet.

At the entrance to the orphanage, there was a cabinet with medications for the children: antibiotics and anti-retrovirals provided by USAID. Above the cabinet there were little urns holding the ashes of the children who had died. Beside each urn was the favorite toy of each child who had succumbed to AIDS: a teddy bear, a toy car. They were like little shrines to the young lives of the children who had lived with their brothers and sisters in their orphan family (see Figure 2).

Many of the orphaned children had parents who contracted HIV through injection drug use. If peeled down to their very core, all approaches to drug dependence: treatment, prevention, harm reduction, treatment and enforcement approaches to drug dependence share a common humanistic element. The healthcare practitioner's commitment to treating the hardest to treat patient, with traditional treatment or harm reduction, is based on love for the patient's humanity and hope for their well-being. Attempts at prevention are based on the devotion of professionals and the eagerness of communities, out of love, to prevent people from drug dependence and its risk. In many cases, the police officer's attempt to stop the import and distribution of drugs may be founded in a devotion to the people and communities that they serve and protect.

The fact that children of the shelter have been failed so miserably by public policies that could have prevented HIV/AIDS continues to haunt me. Many of the parents of these children were injection drug users and survival sex trade workers whose HIV/AIDS could have been prevented with proper access to clean syringes, pharmaceutically assisted therapies, shelter, education, nutrition and healthcare. It seems shameful that prevention, treatment, harm reduction and enforcement systems have failed to adequately protect families and children like those living in Mai Tai shelter from contracting HIV. Victims of stigma and failed public policies, these children are now unwelcome in the wider world: forced to seek shelter outside the mainstream community. In the face of the detached little world of the orphans, I was reminded that there isn't really a world, system or society that could adequately shoulder the blame. Worlds, systems and societies aren't self-determining. They are comprised of the same intentional building blocks: you and I.

The roots of harm reduction and population health are in its attempt at curbing the deleterious effects of HIV, HCV and fatal overdoses. These roots can also be traced to a devotion to people and communities. This theme, kindness and love, comes to life in the orphan community, as the children and their father care for one another, at the Mai Tai shelter. Perhaps, love, itself, is harm reduction.

## Competing interests

The author declares that they have no competing interests.
